# Genetic analysis of the *TMEM230* gene in Chinese Han patients with Parkinson’s disease

**DOI:** 10.1038/s41598-017-01398-9

**Published:** 2017-04-26

**Authors:** Chang-he Shi, Fang Li, Meng-meng Shi, Zhi-hua Yang, Cheng-yuan Mao, Shu-yu Zhang, Hui Wang, Yuan Cheng, Jing Yang, Jun Wu, Yu-ming Xu

**Affiliations:** 1 Department of Neurology, The first affiliated Hospital of Zhengzhou University, Zhengzhou University, Zhengzhou, 450000 Henan China; 2 Institute of Clinical Medicine, The first affiliated Hospital of Zhengzhou University, Zhengzhou University, Zhengzhou, 450000 Henan China

## Abstract

*TMEM230* mutations have been recently reported to cause autosomal dominant Parkinson’s disease (PD). However, there are limited studies from different ethnic populations to support the role of *TMEM230* in sporadic PD. In this study, we performed a comprehensive *TMEM230* mutation screening in 550 sporadic PD patients and 560 controls to elaborate the genetic contribution of *TMEM230* to sporadic PD. Overall, we did not find any pathogenic mutations in the coding sequence, while we identified four variants (c.68 + 182G > A, c.78A > G, c.552 + 11A > G and c.174 + 11C > T) both in the patients and controls, and c.68 + 182G > A appeared to be associated with an increased risk of PD (odds ratio 1.782, 95% confidence interval 1.035–3.067, *p* < 0.05). After Bonferroni correction, however, c. 68 + 182G > A had no significant association with sporadic PD (*p*_c_ = 0.136, *p*_c_ > 0.05). Thus our results suggest that *TMEM230* gene mutations may be rare in Chinese populations, and the variability of *TMEM230* gene may not be a main factor for sporadic PD patients in Chinese Han populations. More evidence is still needed to clarify this question.

## Introduction

Parkinson’s disease (PD) is a progressive neurological disorder affecting approximately 1% of individuals above 65 years of age^1^. The typical motor symptoms include resting tremor, muscle rigidity, bradykinesia and postural instability, which result from the selective degeneration of dopaminergic neurons and axonal projections in the substantia nigra. The exact pathogenic mechanism underlying PD is still elusive. Although inherited cases caused by genetic mutations only account for 10% of PD patients, these genetic findings have provided novel insights into the pathogenesis of PD^2^. Recently, accumulating studies have revealed the association between vesicle trafficking and PD, and evidence from genetic studies revealed that mutations in vesicle trafficking-related genes (*VPS35*, *RAB39B*, and *DNAJC6*) were associated with familial PD^3–5^.

Recently, *TMEM230*, a novel gene involved in vesicle trafficking, was identified to be associated with clinically typical, autosomal dominant and Lewy body–confirmed PD. A missense mutation (c.422G > T; p.Arg141Leu) in *TMEM230* was first identified in a large pedigree from North American and three other mutations (c.551A > G, c.275A > G, and c.550_552delTAGinsCCCGGG) were also detected in other PD patients. Moreover, c.550_552delTAGinsCCCGGG mutation was found in 7 Chinese familial cases^6^. These results suggested that mutations in *TMEM230* gene might be novel genetic causes for PD. However, evidence from recent studies in different populations did not fully conform to these new findings^7–11^.

In an effort to further investigate the relationship between *TMEM230* and sporadic PD in our population, we performed a comprehensive *TMEM230* mutation screening in 550 sporadic PD patients and 560 healthy controls.

## Results

We did not identify any pathogenic mutations in the coding region of *TMEM230* gene in the PD patients and controls, while we identified three known (c.68 + 182G > A, c.78A > G, c.552 + 11A > G) and one unknown SNPs (c.174 + 11C > T) both in the patients and controls (Table 1). Moreover, c.68 + 182G > A appeared to have significantly different frequencies in two groups (odds ratio [OR] 1.782, 95% confidence interval [CI] 1.035–3.067, *p* < 0.05). After Bonferroni correction, however, there was no significant difference for c.68 + 182G > A in genotypic distribution between PD patients and controls (*p*_c_ = 0.136, *p*_*c*_ > 0.05).

**Table 1:** Alternative minor allele frequencies of identified *TMEM230* variants.

Position at chr20	rs number	cDNA	Amino acid	Alternative minor allele frequency		PD vs Controls	
				PD	HC	OR (95% CI)	P*
5112779	rs149865687	c.68 + 182G > A	intron	0.0318	0.0179	1.782 (1.035–3.067)	0.034
5111596	rs745443202	c.78A > G	Leu26Leu	0.0027	0.0009	3.055 (0.318–29.320)	0.308
5111489	novel	c.174 + 11C > T	intron	0.0172	0.0143	1.209 (0.625–2.399)	0.572
5100780	rs750802038	c.552 + 11A > G	3′UTR	0.0045	0.0036	1.273 (0.343–4.727)	0.718

*P Value was determined using the Pearson’s χ^2^ test.

PD: Parkinson’s disease; HC: Healthy control; OR: odds ratio; CI: confidence interval.

## Discussion

*TMEM230* plays a critical role in cellular vesicle dynamics, especially synaptic vesicles. Recently, *TMEM230* has been identified as a disease-causing gene in PD. Moreover, the genetic defect of *TMEM230* discovered by Deng *et al*. has proved to impair the movement of vesicles and lead to the failure of α-Synuclein degeneration^6^. Further functional studies also revealed that TMEM230 not only was involved in retromer trafficking and Rab8a-mediated exophagy and classical secretion but also shared a converging pathway with leucine-rich repeat kinase 2 (LRRK2), which provided several lines of evidence for the association between *TMEM230* and PD pathogenesis^12^. However, subsequent genetic analysis from different ethics found *TMEM230* gene pathogenic mutations were rare both in familial and sporadic PD patients^7–11^. As a result, further genetic investigations are necessary to clarify the role of *TMEM230* in PD.

In this study, we conducted a comprehensive screening of *TMEM230* gene in 550 sporadic patients and 560 controls, and failed to identify any pathogenic mutations in the coding sequence. Our results suggested that *TMEM230* gene mutations were rare in Chinese Han PD patients, at least in our population. Besides, we detected 4 variants (c.68 + 182G > A, c.78A > G, c.174 + 11C > T, c.552 + 11A > G) both in the cases and controls, and c.68 + 182G > A might be associated with an increased risk of PD (OR 1.782, 95% CI 1.035–3.067, *p* < 0.05). After correction for multiple testing, however, allele frequencies of c.68 + 182G > A showed no significant difference in two groups (*p*_*c*_ > 0.05), indicating that c.68 + 182G of *TMEM230* gene might not confer the risk of sporadic PD in Chinese population. Given the limited patients we included, larger samples are needed to testify the role of the variant c.68 + 182G > A in this disease in case of false-negative results.

In conclusion, our results indicate that *TMEM230* mutations are rare in Chinese Han patients with sporadic PD. None of the four variants (c.68 + 182G > A, c.78A > G, c.174 + 11C > T, c.552 + 11A > G) have significant association with Chinese sporadic PD. The variability of *TMEM230* gene may not be linked to sporadic PD patients in Chinese Han populations. Still, further genetic studies including larger sample sizes from different ethnic groups are required to clarify the pathogenic role of *TMEM230* gene in PD.

## Methods

### Patients

A cohort of 550 Chinese Han sporadic PD patients (mean age, 55.9 ± 15.3 years, Male to Female ratio = 319/231) was collected. All the patients were enrolled from the first affiliated Hospital of Zhengzhou University between 2010 and 2016. All patients were submitted to a standardized neurological examination by two movement disorder specialists, and the diagnosis adopted by the doctors was made according to the criteria of the United Kingdom PD Society Brain Bank. The control group was consisted of 560 age and sex matched healthy individuals (mean age, 53.7 ± 14.9 years, Male to Female ratio = 308/252) from the same geographic areas. The study was approved by the Ethics Committee of First Affiliated Hospital of Zhengzhou University and informed consent was obtained from all the participating subjects. All experiments were performed in accordance with the approved guidelines.

### Mutation Analysis

Genomic DNA was extracted from peripheral blood collected from the patients and controls using standard protocols. The entire *TMEM230* coding region and exon-intron boundaries were sequenced from genomic DNA. Polymerase chain reaction (PCR) analysis of the *TMEM230* gene was carried out using primer pairs described previously^6^. Both cases and controls were genotyped by Sanger sequencing. DNASTAR Lasergene MegAlign (v7.1.0) and Chromas (v2.33) were used to conduct sequence alignment.

### Statistical Analysis

We did a case-control study using the data which was detected in PD patients and normal controls. Allele frequencies in case and control subjects were compared using Pearson’s χ^2^, and we used Bonferroni correction to adjust for multiple testing. We also calculated ORs and 95% CI of minor alleles found in this study. *p* value of 0.05 was regarded as statistically significant.
